# Human population history on the North Coast of Peru from Y chromosomes and mitogenomes

**DOI:** 10.1038/s41598-025-08241-6

**Published:** 2025-07-27

**Authors:** Lea Lorene Huber, Epifanía Arango-Isaza, José R. Sandoval, Matthias Urban, Paolo Francalacci, Carla Calò, Enrico Macholdt, Mark Stoneking, Lutz Roewer, Maria Seidel, Oscar Acosta, Ricardo Fujita, Kentaro K. Shimizu, Chiara Barbieri

**Affiliations:** 1 Department of Evolutionary Biology and Environmental Studies, University of Zurich, 8057 Zurich, Switzerland; 2 Department of Biology, Aarhus University, Aarhus, Denmark; 3 Centro de Genética y Biología Molecular, Facultad de Medicina, Universidad de San Martín de Porres, Lima, Perú; 4 Centre National de La Recherche Scientifique Laboratoire “Dynamique du Langage” (UMR 5596), Lumière University, Lyon 2, Lyon, France; 5 Department of Life and Environmental Sciences, University of Cagliari, 09126 Cagliari, Italy; 6 Department of Evolutionary Genetics, Max Planck Institute for Evolutionary Anthropology, Leipzig, Germany; 7 Biométrie Et Biologie Évolutive, UMR 5558, CNRS &, Université de Lyon, Lyon, France; 8 Institute of Legal Medicine and Forensic Sciences, Department of Forensic Genetics, Charité Universitätsmedizin Berlin, Berlin, Germany; 9 Institute of Legal Medicine, Department of Forensic Genetics, Universitätsklinikum Schleswig-Holstein, Kiel, Germany; 10 Kihara Institute for Biological Research, Yokohama City University, Yokohama, 244-0813 Japan

**Keywords:** South American history, mtDNA, Short Tandem Repeats, aDNA, Haplogroups, Moche culture, Population genetics, Archaeology

## Abstract

The Central Andes and Pacific coast of Peru were an important center of cultural development in prehistoric South America. In particular, the North Coast of Peru had a significant demographic weight and witnessed a succession of societies and polities, some of which achieved state-level complexity. To understand the impact of this legacy on the genetic diversity of people living today, we generated 76 Y-chromosomal STR profiles and 143 full mtDNA sequences from four communities of the Peruvian North Coast. We reconstruct genealogical trajectories and search for connections to other living populations from South America, as well as with ancient individuals from archaeological contexts. We find characteristic paternal and maternal lineages, found only in the North Coast. These distinct genetic profiles are deeply rooted, and some of them can be linked with ancient individuals from local archaeological sites such as La Galgada (4000 years ago), and Moche sites like El Brujo (1600 years ago) and Huaca Prieta (1400 years ago). Additionally, a north–south divide from haplotype sharing profiles partly mirrors archaeological and linguistic dissimilarities already present at the time of the Moche culture. The multidisciplinary evidence examined suggests that the demographic distinctiveness of the North Coast populations of Peru is paired by exchanges with neighboring Peruvian and Ecuadorian groups and a high intrapopulation diversity.

## Introduction

Within South America, the Central Andes represent an important cultural area that gave rise to the most prominent South American state-level societies. The region, which comprises most of present-day Peru and the Bolivian parts of the Lake Titicaca basin, is split into three ecological domains that influence the local way of living: the Andean highlands, the Pacific Coast, and the eastern slopes of the Andes. These host three recognized centers of cultural development: the North Coast, the southern highlands of Peru, and the Titicaca basin^1^. Ancient societies of the Central Andes were based on intensive and specialized cultivation. On the North Coast, they developed a system of canal irrigation to transform the hyperarid desert into fertile land^2–4^, and they also practiced fishing which played a significant role in their economy^5^. In the southern highlands, where specialized cultivars were adapted to different altitude levels, large settlements and complex societies including Tiwanaku, Wari and the short-lived Inca Empire appeared slightly later than on the coast^1,6,7^.

The North Coast has received relatively less attention from geneticists and from the general public, compared with the southern highlands and its most renowned empires revolving around Cuzco and the Titicaca Lake – despite the well-grounded archaeological research. The first coastal culture generally considered to have achieved state-level complexity is the Moche culture, which emerged in the first half of the 1^st^ millennium CE^8^. The Moche built pyramids and produced ceramics with a highly distinctive style, featuring representations of individuals, animals, cultivated species, erotic imagery, and detailed scenes of ritualistic behavior, reflecting an elaborate cosmological vision^9–11^. The Moche area can be divided at the Pampa de Paiján into a northern and a southern sphere, each with distinct cultural expressions and political organization. Moche elites were in contact with neighboring cultures both along the southern coast (Lima, Pachacamac, Nazca) and in the highlands (Recuay, Chachapoya, Cajamarca). Around 600 CE the ceramic style associated with the Moche gradually disappeared, although their culture persisted until 800 CE, giving way in the northern sphere to the Sicán or Lambayeque cultural complex. Sicán society was stratified and probably multi-ethnic; its trade network expanded north into Colombia and east past the Andes into the Amazonian lowlands^12^. This culture collapsed around 1100 CE, making way for an expansion of the Chimú culture which developed to the south. The Chimú state, known as Chimor, was expansive and powerful; its capital Chan Chan, near the present-day town of Magdalena de Cao, was one of the largest urban centers of the Americas, inhabited by ~ 50,000 people^16^. The Chimor zone of influence reached from the very north of Peru (including Tumbes and Piura) down to Lima, but not into the highlands. Chimor was conquered by the expanding Inca empire about 60 years before the arrival of the Spanish. The Inca rulers are known to have forcefully relocated populations from different corners of the empires, a practice called *mitmaq*^17^; however, it is unclear to what extent this practice disrupted local social and power structures. The Spanish occupation then had a profound effect, bringing diseases, forced labor practices and violence, which led to a collapse of native populations and the erasure of many indigenous cultural practices and languages^18^. Moreover, colonial structures established since the sixteenth century continue to influence present-day society. These dramatic changes have made it challenging for researchers to access and understand pre-colonial history and societal dynamics.

Archaeology informs about material culture and allows inferences about the societies that created them. Linguistics can be integrated to assess the degree of cultural differentiation within the region, for example through the study of toponyms and from early Spanish sources on the linguistic situation. The linguistic diversity in the North Coast was high; at least four languages *–* Mochica, Quingnam, Tallán, Sechura – were spoken^13^*.* Of these now-extinct languages Mochica survived the longest, and was still spoken in the fisherman village of Eten in the early twentieth century^13^. The languages are poorly documented, and the available documentation does not suggest the presence of language family relationships between themselves, nor with any other known South American language family^19,20^. However, the languages of Northern Peru were characterized by specific features such as sounds, word structure and vocabulary items that are not known in the southern half of the Central Andes, where the Quechuan and Aymaran language families were (and still are) spoken. These similarities suggest relatively intense contact relationships between speakers of the languages in prehistory^13,19,21^.

To complement the historical and archaeological reconstructions, it is important to verify demographic aspects associated with the cultural and political successions: continuity through time, levels of interaction, and variation in population size. Genetic studies are a powerful tool for this purpose. The first migrants who left a genetic trace in the current population of the Americas entered through a coastal route about 16 to maximally 19.5 kya, with a rapid spread along the continent^22,23^. In South America, three major ancestries are detected: one primarily found in Amazonia, one primarily found in the Southern Cone, and one primarily found on the Pacific Coast and in the Andes^24^. The coastal populations are diverse and closely connected to the Central or North-Eastern Andes of Peru^25^. This supports a coastal immigration route with subsequent colonization of the Andes from the Coast^26^, but could also be a sign of continuing intense contact. However, the coastal populations are distinguishable from the highland populations on a finer scale^20,25,27^. The higher genetic diversity and larger population sizes of coastal and Andean populations when compared to Amazonian populations suggest the persistent presence of large societies^28,29^, as supported by the archaeological record. Populations from the southern Central Andes are intensely intermixed and genetically more homogeneous than those in the northern Central Andes^25,30^*.*

Ancient DNA (aDNA) from archaeological sites can be compared to the genetic diversity of living populations, anchoring the genetic reconstructions through time. Analysis of aDNA from the Central Andes has highlighted the formation and persistence of genetic structure^31–33^. Continuity was high and population displacement was probably not extensive in the last 2000 years^20,27^. Forceful relocations by the Inca (*mitmaq)* did not disrupt the genetic landscape: they have been tracked with genetic data in some cases^33^, but their portrayal by early Spanish chronicles was possibly exaggerated^34^.

In this study, we utilize uniparental markers: paternally inherited Y chromosome and maternally inherited mtDNA, for which large comparative published data from South America is available, for both living and ancient individuals (see methods). We generated Y chromosome short tandem repeats (STRs) haplotypes for 76 males, and full mtDNA genomes for 143 individuals from four locations of the North Coast of Peru, selecting individuals belonging to characteristic Native American haplogroups for our analysis. A subset of these samples has been previously analyzed with genomic SNP chip data in a large-scale genetic study of living populations of Western South America^25^. In this study, instead, we focus on the Peruvian North Coast and on the comparison between ancient and modern DNA. With fine-grained genealogical comparisons, we describe the genetic history of this region and contextualize its relationships with the Andes and nearby Amazonia. Our results complement the reconstruction of population history in regions where written historical reports are scanty.

## Results

**Fig. 1:**
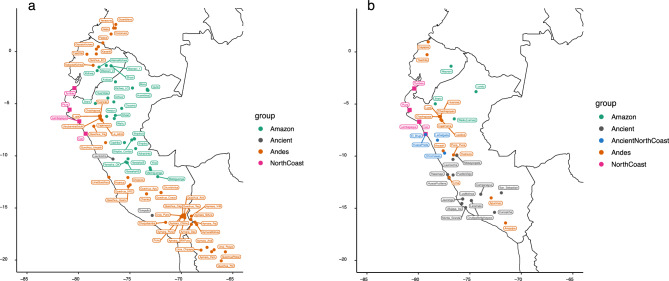
Location of the four target populations of the North Coast of Peru (pink squares) within the range of Western South American (Central Andes) samples. (**A**). Subset of populations included in Y chromosome analyses. (**B**). Subset of populations and individual profiles included in mtDNA analyses.

To focus on the prehispanic history of the continent, we consider only the haplogroups of characteristic Native American origin: haplogroup Q for the Y chromosome (without considering the rare haplogroup C3-MPB373) and haplogroups A2, B2, C1b, C1c, C1d1, D1 and D4h3a (without considering the rare haplogroup X) for the mtDNA^45^ – see Methods. For these haplogroups, we genotyped 17 STR Y chromosome loci and sequenced full mtDNA genomes (Supplementary Table S1). The participants come from four locations of the North Coast of Peru at different latitudes: Tumbes, Piura, Lambayeque, and Cao (Fig. 1).

### Paternal genetic profiles

**Fig. 2:**
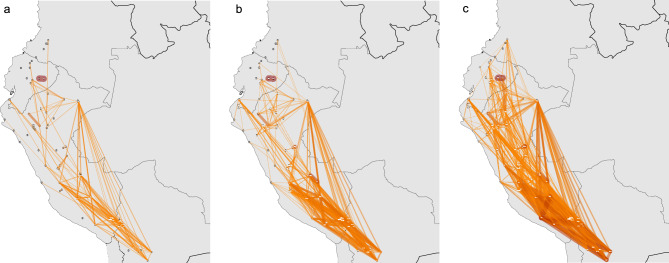
Maps of haplotype sharing between populations in the Central Andes. (**A**). frequency of sharing of identical haplotypes. (**B**)**.** frequency of sharing of haplotypes with an estimated tMRCA of < 500. (**C**). frequency of sharing of haplotypes with an estimated tMRCA of < 1000 years ago. In B. and C., only continent-wide above median frequencies are plotted. The thicker and darker the line connecting two populations is, the more frequently do they share identical/similar haplotypes. Unconnected populations do not share any identical/similar haplotypes or do so with a frequency below the median.

We analyzed the male demographic history of the North Coast populations both at the individual and at the population level, with a large continental comparative dataset (Supplementary Fig. S1, Supplementary Table S2 and S3). At the individual level, we compared haplotype distances using an approximation to the time to the most recent common ancestor (tMRCA), using bins of 2000 years ago (a time roughly corresponding to the emergence of the Moche culture), 1000 years ago (development of Sicán and early Chimú before expansive imperial phase), and 500 years ago (expansion of the Inca empire). The oldest time frame is reconstructed with less precision, and sees a natural increase of shared ancestries through broader regions. Pairs of populations with a high frequency of a tMRCA < 2000 years are found within (east) Amazonian populations, within Andean populations (especially the Aymara and Quechua populations), and within Mesoamerican populations (Supplementary Fig. S2). Populations of the North Coast share most ancestry with each other, in all timespans (Supplementary Fig. S3). Recent (< 500 y) tMRCAs between the North Coast and other regions are rare. However, appreciable frequencies of tMRCA < 2000 years and < 1000 years can be observed between the North Coast and the Andes and between the North Coast and the Amazon, which are additionally the only regions that share identical haplotypes with the North Coast. The maps in Fig. 2 show pairwise sharing of identical and similar haplotypes restricted to populations from the focus region of the Central Andes. Haplotype sharing within the North Coast populations is not pronounced, compared to the intense haplotype sharing within southern Central Andean populations. Nonetheless, there is appreciable sharing between Lambayeque, Piura, and Tumbes. Cao does not share identical or similar haplotypes with the other North Coast populations, nor does it share identical haplotypes with any other population in the dataset. Tumbes is the most connected of the four North Coast populations: it shares identical haplotypes with Quechua populations and other populations in the southern Central Andes (Huanca, Aymara, Potosi) as well as ancient Candelaria. Piura shares identical haplotypes with Jivaro, Quechuans from Cajamarca, and the ancient Lauricocha site. Within the last 500 years (Fig. 2B), based on sharing patterns with an appreciable frequency above the median, Lambayeque is connected with Utcubamba, Shawi and Candelaria; Tumbes shares common ancestry with various Aymara and other southern Central Andean populations, Llanos from Pando, Iquito and Machiguenga (Amazon); and Cao with Quechua speaking populations from the central Andes. In the last 1000 years, patterns of shared ancestries became broader (Fig. 2C), connecting Cao to the Amazon, Tumbes to the north (with populations from Colombia), and connecting Tumbes, Piura, Lambayeque together with various populations of the southern Central Andes.

**Fig. 3:**
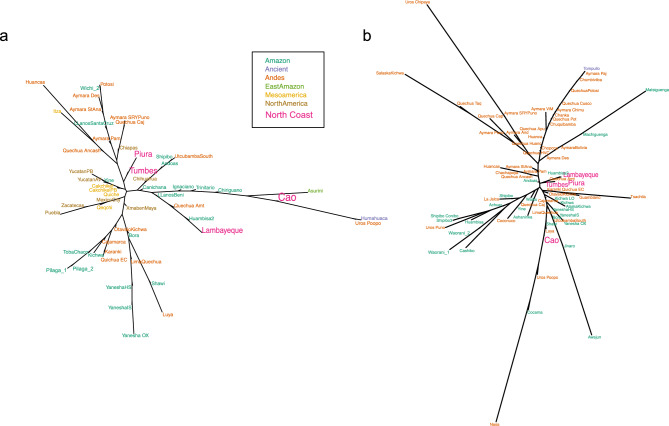
NJ trees from R_ST_ distances calculated with 17 STR loci. North Coast populations are highlighted with a larger font. (**A**). subset of all the populations from the continental dataset, including those with an R_ST_ < 0.03 to each North Coast population. (**B**). subset of the populations from the Central Andes of Western South America, as included on the map in Fig. 1A.

We computed R_ST_ population pairwise distances and visualized them with a NJ tree (Fig. 3) and with a heatmap (Supplementary Fig. S4). The North Coast populations are not particularly distinct, showing low R_ST_ distances to most populations of the dataset. They do not display a particular tendency to be grouped either with Andean or with Amazonian populations (Supplementary Fig. S4). The NJ tree (Fig. 3) highlights the relatedness between Tumbes and Piura, which are also loosely related to Lambayeque in Supplementary Figure S4, with low distances to various Andean Quechua and Aymara populations. Cao is more distinct, close to populations from Amazonia (including Trinitario, Chiriguano, Asurini, and Utcubamba). Lambayeque is relatively close to a population from the Peruvian Amazon (Huambisa). The specific relatedness of the North Coast populations is more discernible when only the populations of the Central Andes are compared (Fig. 3B).

We finally examined parameters of population diversity to quantify the degree of isolation and consanguinity in comparison to other South American populations (Table 1, Supplementary Fig. S5). Haplotype diversity is high in the four populations, ranging from 0.98 in Lambayeque and Piura to 1 in Tumbes and Cao (with 1 corresponding to each individual of the population carrying a different haplotype). Haplotype variance measures how diverse non-identical haplotypes are; high values can indicate the presence of substructure within a population. Of the North Coast populations, Cao has the highest variance, indeed the second highest value over all populations in the dataset (0.96). Compared to all populations, Tumbes has an intermediate variance (0.61), while the variance values for Lambayeque and Piura are in the lower half (0.54 and 0.50 respectively). These values are in line with those from other populations from Western South America (Supplementary Fig. S5). Within the Central Andes, there is no clear discernible trend; variance rather seems to be a trait that varies between individual populations.

**Table 1:**
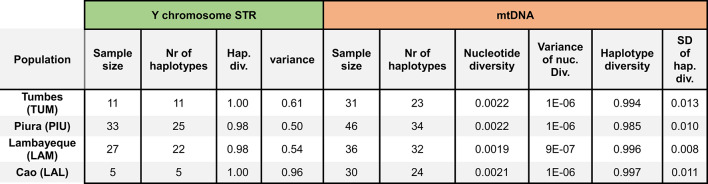
Sample size and population diversity values for the four newly genotyped populations from the North Coast.

We then focused on the four North Coast populations, utilizing the full dataset of 23 Y chromosome loci, to investigate relationships at a finer scale. We generated a heat map of individual distances between all pairs of individuals from the North Coast (Supplementary Fig. S6). A few population-specific branches of closely related haplotypes are visible: one with 12 individuals from Lambayeque (plus one individual from Tumbes); two with seven individuals from Piura and three individuals from Lambayeque; and another one with again seven from Piura and two individuals from Tumbes. The five individuals from Cao are not particularly close to each other.

### Maternal genetic profiles

**Fig. 4:**
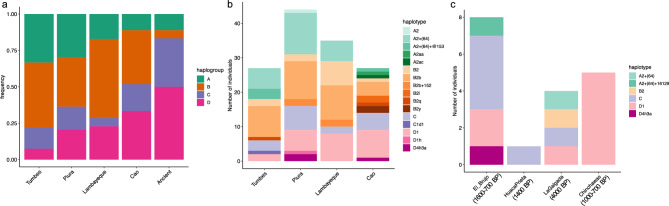
Haplogroups and sub-haplogroups in the modern and ancient North Coast area. (**A**). Frequency of the four major native American mtDNA haplogroups in the newly sequenced North Coast populations (ordered from north to south) and from all the ancient North Coast individuals from archaeological sites grouped together. (**B**). number of individuals belonging to each haplotype in the modern North Coast populations. (**C**). number of individuals belonging to each haplotype in the archaeological sites available, dated roughly between 4000 and 700 BP. Sub-haplogroups (or haplotypes) were assigned with Haplogrep^47^, based on Phylotree v.17-FU1^48^.

We first examined haplogroup and sub-haplogroup frequencies of the North Coast. Of the 143 individuals sequenced, nine were assigned to L1, L2, and L3 haplogroups (frequent in Africa) and one to haplogroup H6 (frequent in Europe). The remaining 133 individuals belonging to Native American haplogroups A, B, C, and D were considered for the analysis. The four populations from the North Coast differ from each other in their relative mtDNA haplogroup frequencies (Fig. 4A), with less haplogroup A in the southern population of Cao, and less haplogroup D in the northern population of Tumbes. Mitochondrial comparisons with published aDNA are particularly relevant, as good quality ancient mtDNA has been sequenced from archaeological sites in the Central Andes and in the North Coast in particular. We compared haplogroups from the present-day North Coast against those retrieved from five neighboring archaeological excavations. Common sub-haplogroups between modern and ancient DNA from the North Coast correspond to generic D1 and C haplogroups, without further downstream specifications (Fig. 4B, C). The major haplogroup sub-branches already present in the ancient individuals are still represented in today’s populations, except A2 + (64) + 16,129. Haplogroup B is the rarest in the ancient individuals, while C and D are the most common (Fig. 4C). It was previously noted that haplogroup B increases in frequency in the region only from the Late Intermediate Period^46^.

We calculated individual diversity measures for the four North Coast populations (Table 1, Supplementary Table 4). Lambayeque has the least nucleotide diversity, while Piura, which displays the most nucleotide diversity, has the lowest haplotype diversity. The values are overall similar to those calculated for mtDNA genomes in other north Peruvian populations^34^. The average pairwise distances between our four populations (Supplementary Table 4) show that Cao and Tumbes, the most geographically distant populations, are also genetically the most distant. Between North Coast populations, Tumbes and Piura share the largest amount of identical haplotypes (12 haplotypes, including six identical haplotypes in individuals from Piura), compared to one shared haplotype between Piura and Lambayeque and two between Tumbes and Lambayeque (relative frequencies in Supplementary Table 4).

**Fig. 5:**
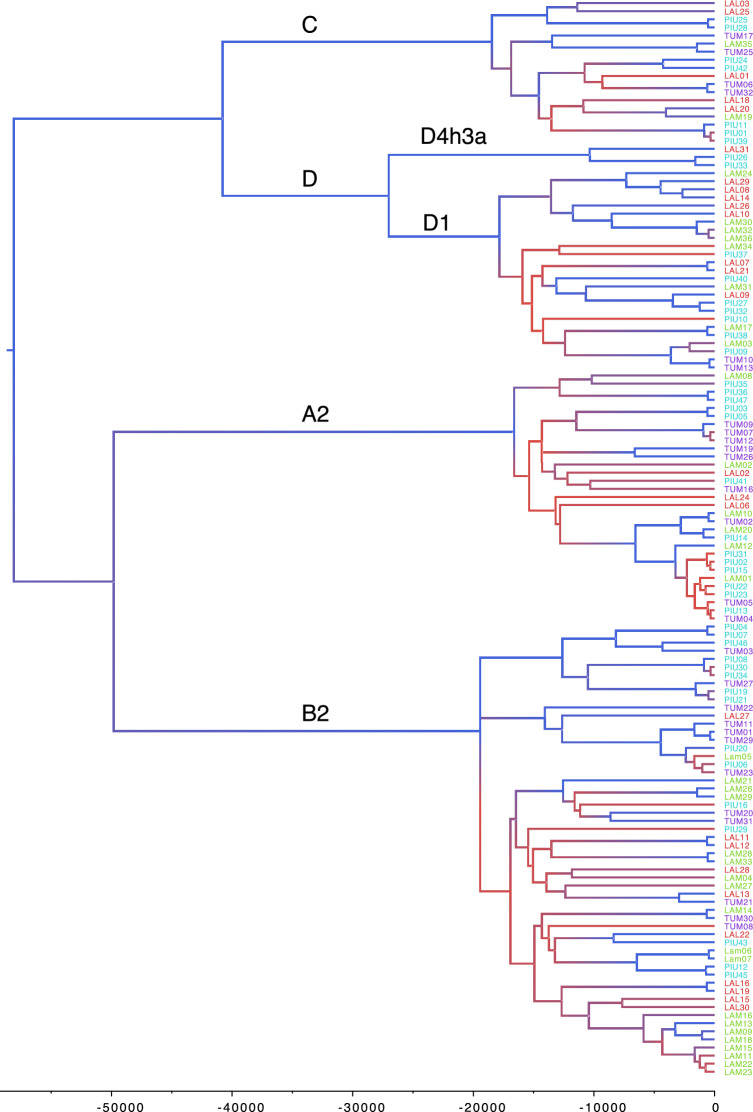
Bayesian phylogenetic tree of mtDNA sequences from the North Coast individuals belonging to one of the 4 major native American mtDNA haplogroups, generated with BEAST and calibrated with mutation rates from Soares et al. ^50^. The individuals are color coded according to their population. The horizontal axis shows the time before present in years. The branches are colored according to the posterior probability value, high posteriors are in blue and low posteriors in red.

Relationships between the mtDNA sequences from the North Coast are displayed in a time-calibrated Bayesian phylogenetic tree (Fig. 5). The four Native American haplogroups are clearly separated with each branch coalescing between 40 and 50 kya, and the branch of D4h3a diverges from D1 at about 27 kya. Between about 17 and 11 kya, i.e. about the time of the colonization of the Americas and subsequent population expansion, lineages diversify quickly, resulting in possible polytomies that are difficult to resolve (red colored branches in Fig. 5 corresponding to low posterior values). The corresponding Bayesian Skyline plot (Supplementary Fig. S7) shows an increase in effective population size (N_e_) from values of about 1000 at 45 kya, and a peak of 20,000 individuals after the rapid increase which started at about 14 kya. The peak of N_e_ is then followed by a decrease in the last 2000 years to about 7000. These N_e_ numbers are lower than the ones found in the ancient and modern continental comparisons of Llamas et al.^22^, higher than those retrieved for populations from neighboring Chachapoyas of the northern Peruvian Andes^34^, and similar to those found from broad sampling locations from neighboring regions in Ecuador^49^.

To compare the mitogenomes of the North Coast to the rest of the continent, we focused on individual relationships. Contrary to the Y chromosome set, available full mtDNA sequences for the Americas are biased towards studies of single lineages rather than samples representative of a population’s diversity. A list of the sequences used for the continental scale comparisons is available in Supplementary Table 5. We found sharing of identical mtDNA sequences between individuals from the North Coast and other populations from Ecuador and Peru, in particular in the areas of Chachapoyas, Cajamarca and Ancash. We compared the time-calibrated detailed phylogeny from Fig. 5 to the full continental mtDNA genome dataset, computing a NJ tree based on sequence distances (Supplementary Fig. S8). Regional-specific clades, containing only sequences from one of the North Coast populations, can be associated with instances of long-lasting population continuity and regional structure. Several of these population-specific branches coalesce between 8,000 and 4,000 years ago. When these branches are localized in the continental NJ tree we see proximity with individuals from nearby regions: Piura with Ecuador (with PIU24 and PIU42 coalescing at 4269 ya (95% CI [849, 8143]); Cao with Ecuador and northern highlands-Chachapoyas (with LAL29, LAL08 and LAL14 coalescing at 4476 ya (95% CI [1311, 7859]); Tumbes with Ecuador and Colombia (Putumayo) (with TUM19 and TUM26, coalescing about 6600 ya (95% CI [2800, 11,000]). A remarkable example of a North Coast population-specific signal is a branch at the bottom of the haplogroup B clade in Fig. 5, with individuals from only Lambayeque; these sequences coalesce at 4319 ya, and do not show relationships with any other individual of the continental dataset (Supplementary Fig. S8). Broader North Coast-specific branches are also found in the continental NJ trees (Supplementary Fig. S8). A large branch of haplogroup A (from LAM10 to TUM04 in Fig. 5), coalescing at 6500 ya (95% CI 3071 to 10,300 ya), is consistently found in the NJ tree separated from other South American sequences. This North Coast-specific branch does not contain samples from Cao. The only related sample to this branch is an ancient sample from 4000 BP from La Galgada (KU523271), a site in the North Central Andean highlands. We find more examples of North Coast-specific subbranches which do not share similarities with other South American sequences: for example, PIU46 and TUM03 in haplogroup B2 diverge 4318 ya; four sequences from Tumbes (TUM01a, TUM11, TUM23 and TUM29, two from Piura (PIU06 and PIU20) and LAM05 coalesces 4472 ya; PIU27, PIU32 and LAL09 coalesce at 3448 ya; and LAM03, PIU09, TUM10 and TUM13 coalesce at 3646 ya. Generally, we see that North Coast-specific branches coalesce not earlier than 4000 years ago. Overall, there is quite some continuity within populations over the last 2000 years, with very few coalescent events between different populations, and none with Cao: the most recent coalescence of a Cao individual with an individual of another North Coast population was 2943 ya (95% CI [458, 5980]) (LAL13 and TUM21).

**Fig. 6:**
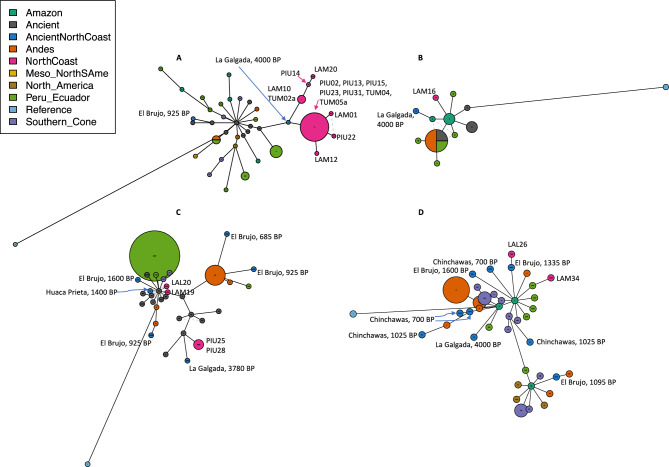
Networks of mtDNA haplotypes. Each network includes a subset of the closest haplotypes to any of the ancient Coast samples. Arbitrary thresholds were chosen to reduce the number of haplotypes in the network for legibility. Networks (**A**–**D**) correspond to the haplotypes belonging to the haplogroups A-D, respectively. The lengths of the connections between haplotypes corresponds to the number of mutation steps between them. The size of the circles corresponds to the number of individuals for each haplotype. North Coast individuals are labelled with their ID. Ancient North Coast individuals are labelled with their archaeological site and age.

We finally analyzed ancient North Coast samples to search for relatedness with living populations. The ancient sites considered in this study are marked in Fig. 1B. Ancient samples and their related sequences can be localized in the continental NJ tree in Supplementary Figure S8. To clarify haplotype relationships between those ancient individuals and their closest relatives, we generated a network for each haplogroup (with a selection of closest haplotypes, in Fig. 6). The most interesting result is found within haplogroup A, and concerns the sample from La Galgada, Kotosh, 4000 BP (KU523271) which is confirmed to be closely related to 14 individuals from the three northern populations of the North Coast (excluding Cao). In haplogroup B, the individual from La Galgada, Kotosh, 4000 BP (KU523310), Kotosh, 4000 BP) is close to Amazonian individuals and one individual from Lambayeque. The same individual is also related to two individuals from Cao in the NJ tree of Supplementary Figure S8. In haplogroup C, an individual from La Galgada (3780 BP) is close to an individual from a Paracas site on the South Coast (Jauranga 1650 BP) and to two individuals from Piura. Four further ancient North Coast samples from El Brujo and Huaca Prieta are loosely related to two North Coast individuals (from Lambayeque and Cao), as well as to other ancient individuals. In haplogroup D, the ancient North Coast samples are loosely related to Andean modern samples, except one sample from El Brujo (1335 BP) which is close to a sample from Cao.

## Discussion

We examined the maternal and paternal genetic profiles of individuals from fishing villages on the North Coast of Peru, grouped into four populations. Subtle differences can be described between the four populations, particularly the genetic distinctiveness of Cao compared to the other three North Coast populations to the north. Based on the Y chromosome haplotypes, the five individuals from Cao do not share recent ancestries with the other North Coast populations in the last 1000 years (Fig. 2); similarly, Cao does not share any identical mtDNA haplotypes with other North Coast populations (see Table S4), while the other three populations all do share haplotypes among each other, indicating more recent shared ancestry and/or contact. Absence of recent sharing is also reflected in the phylogenetic tree of the North Coast mitogenomes (Fig. 5), where the most recent coalescence of a Cao sample with another North Coast sample was almost 3000 ya.

These results partially contradict archaeological and cultural chronologies, as the populations from the Far North (Piura and Tumbes) had ties with cultures of present-day Ecuador and remained for a long time outside the influences of the Central Andean culture area. However, interaction and perhaps mobility increased from late Moche times onward, when people culturally affiliated with the Moche established a presence in the Upper Piura valley^51^ and iconographic evidence from the northern Moche area depicts female foreigners with supposedly northern attributes^52^. Still later, the coastal areas of the Far North also came under increasing influence or even control of North Coast states^53,54^. The unexpectedly strong genetic ties of the Far North with the Lambayeque region suggest connections were significant and had lasting effects on demographic structure.

The relative isolation of Cao from Lambayeque is additionally confirmed by autosomal data^25^: Cao did not share autosomal haplotypes (Identity-By-Descent segments) with the other North Coast populations, and displayed an ancestry component in an admixture analysis which is present at high frequencies in more southern Andean regions like Cuzco. This genetic divide mirrors a cultural divide between a Mochica-speaking area in the north and a Quingnam-speaking area in the south at the Pampa de Paijan. The cultural separation probably took place between 300–400 CE^8,55^, i.e. in a timeframe well detectable with STR analysis. However, the genetic distinctiveness of Cao, contrasting the Lambayecan affinities with the Far North, does not mirror its central role in the emergence of the cultural traditions which then radiated out to more peripheral zones like the Far North. Cautionary notes should accompany these suggestions: first, the small sample size for the Y chromosome analysis, and second, the recent historical migrations motivated by economic reasons of people working in the sugar cane fields. Participants reported family ties to neighboring mountain regions (Sierra La Libertad), which could have introduced foreign haplotypes. Despite the similarity between the three northern populations of Tumbes, Piura and Lambayeque, there is also evidence for population-specific lineages. This is supported by a higher frequency of identical mtDNA haplotype sharing within populations compared to between populations (Table S4) and from similar Y chromosome haplotypes within populations (Supplementary Fig. S6). For the Y chromosome, the sharing of haplotypes between the North Coast populations is not very pronounced, contrary to their autosomal IBD sharing profiles^25^. For the mtDNA, it should be noted that haplotypes could be less diverse due to the lower mutation rate in comparison to the highly polymorphic STR loci, and moreover that the mtDNA comparative dataset has fewer individuals. All sub-branches of mtDNA haplogroups found in the ancient samples are still represented in the present-day populations (Fig. 4B,C). This effect could be due to the permanence of individuals with shared distant maternal ancestries in the same region over time. The presence of local continuity matches the time window of the past 2000 years, confirming previous results for ancient and modern genomic data^27^. Long-term occupation and genealogical continuity is matched by sharing of identical or very similar mtDNA sequences between ancient individuals from archaeological sites and individuals from the present dataset (see Fig. 6). The single exception of the Lambayeque individuals grouped together within haplogroup B could be indicative of earlier sedentariness; however, such a group could also result from incomplete sampling. Earlier than 4000 years ago, branches containing samples only from the North Coast region become exceptions.

The most remarkable case of broad local ancestry through time is the genetic proximity of one haplogroup A individual (KU523271) from La Galgada, Kotosh, 4000 BP, to 14 individuals from the three northern locations of our newly reported sample (Fig. 6A). The La Galgada site is in the highlands, slightly south of Cao, and used to be an administrative and ceremonial place associated with the Kotosh culture, who built monuments of stone there^56^. Direct demographic or cultural connections with the coast are not immediate: for example, the architecture at La Galgada was different from those of contemporary coastal sites^1^. While this connection is relevant, it could be the result of unspecific continuity through time of widespread mtDNA lineages, not exclusive to one specific culture, or it could be a genetic connection mediated by other intermediate shared maternal ancestries. More ancient data from the Preceramic could assess the significance of this finding and contextualize it with other persistent genealogical connections through time transects.

In the sites of El Brujo and Huaca Prieta, we find evidence of genetic continuity with the individuals from the neighboring village of Cao, suggesting continuity in a relatively small area. The site of El Brujo has aDNA for 8 individuals from 1600 – 685 BP, and Huaca Prieta has aDNA for one individual from 935 BP. El Brujo is an archaeological complex located directly on the coast, associated mostly with the Moche, but also with the subsequent Sicán and Chimú cultures^57,58^. The samples were taken from burials associated with Moche and Sicán^27^. Part of the complex is the pyramid of Huaca Prieta, from which one ancient sample is available. Pyramid burials were reserved for those of high social status, while the other individuals buried at El Brujo were likely of lower social status. We find that the haplotype of one ancient individual from El Brujo differs from an individual from Cao in haplogroup D by only one mutation (Fig. 6D), and two individuals from Cao and Lambayeque are more distantly connected to two ancient individuals from El Brujo and Huaca Prieta in haplogroup C (Fig. 6C).

The continental-scale analysis of paternal STR data, which was conducted with a broader continental population-based dataset, did not reveal very specific ties between the North Coast and other populations of the continent, and their connections to other regions seem to be equally diverse. The only exception is Tumbes in the very north of the North Coast, which shows specific recent contacts to the southern highlands of the Central Andes with one haplotype identical to that found in 24 Quechua and Aymara speakers (Fig. 2). The Inca established an important imperial outpost in the Tumbes region before the Spanish conquest^59^; this signal could reflect the presence of administrators and/or *mitmaq* from the imperial heartland. While the connections between North Coast populations and Central Andean populations are not particularly strong, the connections and shared ancestries with other Andean populations are noticeable, especially in the South-Central Andes. Here haplotype sharing patterns suggest significant recent (≤ 1000 y) mobility between populations (see Fig. 2 and Supplementary Fig. S4), while the North Coast populations participate in recent gene flow to a lesser extent. The population fragmentation in the north of the Central Andes as opposed to the high level of interconnections in the south is in line with archaeological findings and mirrors the much more fine-grained linguistic landscape in the north of Peru compared to the south^20^.

The high genetic diversity of the North Coast, together with the presence of regional-specific haplotypes, is in line with a demographic history characterized by large population sizes, which was also detected with ancient^27^ and modern^25^ autosomal profiles. This could also explain the lack of obvious relatedness with other regions of the continent. Recent migrations into the region could have contributed to the genetic structure, in particular for Cao, which has the highest diversity despite little recent gene flow. Gene flow into Cao could be difficult to pinpoint if the source populations are still unsampled.

In an admixture analysis of autosomal SNP data, populations from the North Coast were found to share ancestry with the North Central Andes populations from the region of Chachapoyas^25^. A split from Amazonian populations was modeled to have taken place between 4 and 7 kya, with migration still taking place after that. While the Y chromosome data does not match this connection, the mtDNA sequences from the North Coast are related to the North Peruvian Andes (Chachapoyas and Cajamarca) and Amazonia (Jivaro and Waiku) (Supplementary Fig. S8), as well as to other sequences of unspecified Peruvian or Ecuadorian origin. This effect might be explained by a sex-biased pattern where males have been more stable in the region, carrying characteristic Y-chromosome profiles, and resulting in an overall lesser degree of between population gene-flow.

## Conclusions

Our genetic results, interpreted with insights from archaeology and linguistics, consolidate a possible population history of the North Coast. The shared ancestry of individuals from the North Coast populations and geographically close Peruvian and Ecuadorian populations supports a common ancestry of (part of) the population from the Central Andes. Distinct pottery styles and cultural artifacts considered in combination with the ~ 4000-year-old maternal regional lineages reported here are a sign of the cultural and demographic distinctness of the North Coast region. However, archaeological and linguistic evidence^13^, confirmed by the autosomal^25^, maternal and paternal genetic diversity, suggests that the North Coast populations were by no means isolated but rather were large societies in contact with neighboring regions. Also, gene flow between neighboring populations within the northern Peruvian Coast region likely contributed to the high diversity observed. Overall, this study showed that uniparental markers can complement and refine the findings from autosomal data and aDNA, and their informativeness over different timeframes provides a powerful tool in the study of the human past.

## Methods

### Dataset acquisition and curation

Saliva samples were obtained from 147 voluntary participants from various rural towns and fisherman villages, which have been grouped into four populations (see Supplementary Table S1). The four populations analyzed in this study are named after a geographic reference (name of town or province) as Cao (short code LAL, Magdalena de Cao in province of La Libertad), Lambayeque (LAM), Piura (PIU) and Tumbes (TUM), and will be referred to as “North Coast” populations (see Fig. 1 for sampling locations). The samples were collected during anthropological fieldwork expeditions in 2015, with informed consent and clearance from the Ethics Committee of the University of San Martín de Porres, Lima (Comité Institucional de Ética en Investigación de la Universidad de San Martín de Porres—Clínica Cada Mujer, Ofício No. 579–2015-CIEI-USMP-CCM, 12/05/2015) (see reference^25^). All methods were performed in accordance with the relevant guidelines and regulations. The sampling strategy covers different archaeological and cultural entities that encompass the history of the region. The Far North is covered by a sample from Tumbes (an ancient Inca town) on the Ecuadorian Border and another from Piura, where the Tallán languages were once spoken. The Northern Moche area is represented by the sample from Lambayeque and the Southern Area by the sample from Magdalena de Cao. In Lambayeque, Mochica was spoken in prehispanic times, while Cao belonged to a zone of overlap in which both Mochica and its southern neighbor, Quingnam, likely associated with the Chimor state, were present.

The samples were described in a previous work, where they were analyzed with the Human Origin genomic SNP chip (~ 500,000 SNPs, Axiom^60^) together with other samples from western South America^25^. Sampling procedure, DNA extraction and ethical permits are described there in more detail. The mtDNA and Y chromosome data is presented and analyzed here for the first time. 143 individuals were sequenced for the mtDNA genome, while 123 individuals (males) were first screened for haplogroup assignation (see Methods), and then 76 individuals belonging to Native American haplogroup Q were genotyped for STR data. Separate analyses are performed here for the Y chromosome and mtDNA datasets.

The data was compared to other uniparental profiles spanning South and Meso-America. For the Y chromosome, only individuals from the native American haplogroup Q were considered, from studies where at least 17 STR loci (in one case 16) were genotyped. Modern samples with more than two missing loci were removed. This resulted in a dataset of 4313 individuals, including both modern (4263) and ancient (50) samples from 168 populations (see Tables S2 and S3). The data was then checked for consistency, and individual outliers showing out-of-range repetitions were manually identified and removed. The sample locations for the Y chromosomal continental dataset are in Supplementary Figure S1. For mtDNA, full-length mitochondrial sequences of the Native American haplogroups A, B, C and D were collected from the literature. Samples with more than 5 missing bases were removed. This resulted in a combined database of 2629 individuals spanning Meso- and South America (including the newly sequenced North Coast populations), of which 241 are ancient individuals (Supplementary Table S5). For aDNA, only datasets authenticated with analysis of deamination profiles were considered.

Some analyses were performed on a subset of populations that are geographically close to the North Coast populations, specifically populations from the Central Andes region (which extends from the border region of Ecuador and Peru to the greater Lake Titicaca basin^20^) (see Fig. 1). These regions were chosen with the previous knowledge in mind that the North Coast is part of the Central Andes cultural sphere by evidence from archaeology^13^.

### Y Chromosome genotyping

The North Coast samples were genotyped with the PowerPlex® Y23 System (Promega, Mannheim, Germany). The data was generated in the laboratories of the Institute of Legal Medicine and Forensic Sciences, Department of Forensic Genetics, Charité – Universitätsmedizin Berlin, Berlin, Germany. STR calling was performed with GeneMapper® ID-X1.1.1. (Life Technologies, Darmstadt, Germany) as described in Barbieri et al. 2017^34^. A first screening of basal SNPs was performed on the total sample of 123 males, to assign broad haplogroups and identify individuals belonging to haplogroup Q (haplogroup assignments are reported in Supplementary Table S1). The screening was performed with a SnapShot Assay, as described in Barbieri et al. 2017^34^.

### Mitochondrial DNA genotyping

Library preparation, sequencing, base-calling and read processing of the new North Coast samples was performed in the laboratories of the Max Planck Institute for Evolutionary Anthropology, as described in Barbieri et al. 2017^34^. 143 individuals were successfully sequenced with mean coverage of 2200X (min 341X, max 5464X—Table S1). Haplogroups of the whole comparative dataset were assigned with Haplogrep 2^47^ using the nomenclature from Phylotree v.17-FU1^48^ (see Supplementary Table S1). Only the sequences assigned to Native American haplogroups A, B, C and D were considered for the analysis, resulting in a set of 133 mtDNA sequences. Sequence alignments were generated using MAFFT with RSRS as a reference^61^. They were manually checked, and the two poly-C regions (np 302–315 and 16,183–16,194) were removed from all alignments. A first alignment was performed for the newly sequenced samples alone. The samples were then merged with the continental dataset (Supplementary Table S4), and separate alignments were performed for haplogroups A, B, C and D.

### Data analysis

Both the STR and the mtDNA data were visualized using the gplots^62^ package for heatmaps and ggplot2^63^ for boxplots and maps. The packages maps, rworldmap, ggmap and geosphere were used for the haplotype sharing maps and other maps^64–66^. The APE^67^ package was used to generate Neighbor Joining (NJ) trees and, for mtDNA, the corresponding pairwise distances between mtDNA sequences. NJ trees were corrected manually for negative edges. For determining the diversity of the North Coast populations and for the network analyses, the package pegas^68^ was used. R scripts for visualization and analysis are available upon requests and can be consulted at https://github.com/chiarabarbieri/Ychromosome_STR.

### Y Chromosome analysis

The variance and haplotype diversities of all populations in the compiled dataset were calculated with R: for variance, with function *var*; for haplotype diversity, with function (1—sum(b))*(SampleSize / (SampleSize -1)) where b is a vector of length = number of haplotypes, and for each haplotype returns (number of individual sharing the haplotype / SampleSize)^2. Pairwise distances between individuals were considered as differences in number of repeats, following a Stepwise Mutation Model. We converted pairwise distances into a rough calculation of the time to the most recent common ancestor (tMRCA) between individuals following the Average Squared Difference model. Mutation rates were obtained from YHRD (https://yhrd.org/pages/resources/mutation_rates)^69^. (Supplementary Table S6). Generation time was assumed to be 30 years. For each pair of individuals, the tMRCA was obtained by computing from the mean of the variance at each locus divided by its mutation rate, and finally multiplied by the generation time, as implemented in R with the following:

varM <—apply(rbind(STRhaplotype1, STRhaplotype2), 2, var, na.rm = TRUE).

PairwiseTMRCA <—mean(varM / mutationRates) * generationyears.

R_ST_ was calculated using an online tool by YHRD (https://yhrd.org/pages/tools/amova)^69^, using subsets of 17 loci, and only complete haplotypes. These reduced datasets exclude all Panamanian populations and some ancient populations that had missing loci, resulting in a dataset of 154 populations and a lower sample size for some other populations. Populations with less than 5 individuals were excluded from subsequent analyses (less than 4 for R_ST_ analyses), except ancient populations. Negative R_ST_ values were manually corrected to 0 before using them to build NJ trees with R function *nj*.

### Mitochondrial DNA analysis

Phylogenetic trees of the North Coast individual sequences were generated with Bayesian statistics using BEAST 2.6.7^70^. The sequences were partitioned into the coding and the non-coding region, for which separate clock and substitution rates were tested. The best substitution model for the partitions was evaluated using bModelTest in BEAST as described in Taming the BEAST^71^. The best clock model was determined via nested model testing of a strict clock against a relaxed uniform clock with log-normal distribution (UCLN) in BEAST.

The best substitution model for the coding region as determined by bModelTest was TN93 + invariable sites + gamma. The best substitution model for the control region available in BEAST was HKY + invariable sites + gamma. Nested model testing resulted in significant support for the strict clock model. BEAST was run using these models, a substitution rate of 1.708E-8 per nucleotide per year for the coding region and 9.883E-8 per nucleotide per year for the control region^50^ and a coalescent Bayesian Skyline tree model^72^ in order to obtain a Bayesian Skyline plot (BSP) for 30 million chains. All the ESS values were above the cut-off of 200 (as determined by Tracer v1.7.2^73^). The resulting trees were combined into a maximum clade credibility (MCC) tree using TreeAnnotator and visualized with FigTree (http://tree.bio.ed.ac.uk/software/figtree/).

## Supplementary Information

Supplementary Information 1.

Supplementary Information 2.

## Data Availability

Newly genotyped Y chromosome haplotypes are available in Supplementary Tables. mtDNA sequences are deposited in GenBank/BankIt with accession numbers PQ827204-PQ827346.
