# Supplementary material for: Human population history on the North Coast of Peru from Y chromosomes and mitogenomes
Source: Sci Rep. 2025 Jul 27;15:27362. doi: 10.1038/s41598-025-08241-6 (PMC12301434; doi:10.1038/s41598-025-08241-6)
Supplement: Supplementary file 2 — Supplementary Information 2. [file 41598_2025_8241_MOESM2_ESM.pdf]

Supplementary Figures

Human population history on the North Coast of Peru from Y chromosomes and mitogenomes

Lea Lorene Huber, Epifanía Arango-Isaza, José R. Sandoval, Matthias Urban, Paolo Francalacci, Carla Calò, Enrico Macholdt, Mark Stoneking, Lutz Roewer, Maria Seidel, Oscar Acosta, Ricardo Fujita, Kentaro K. Shimizu, Chiara Barbieri

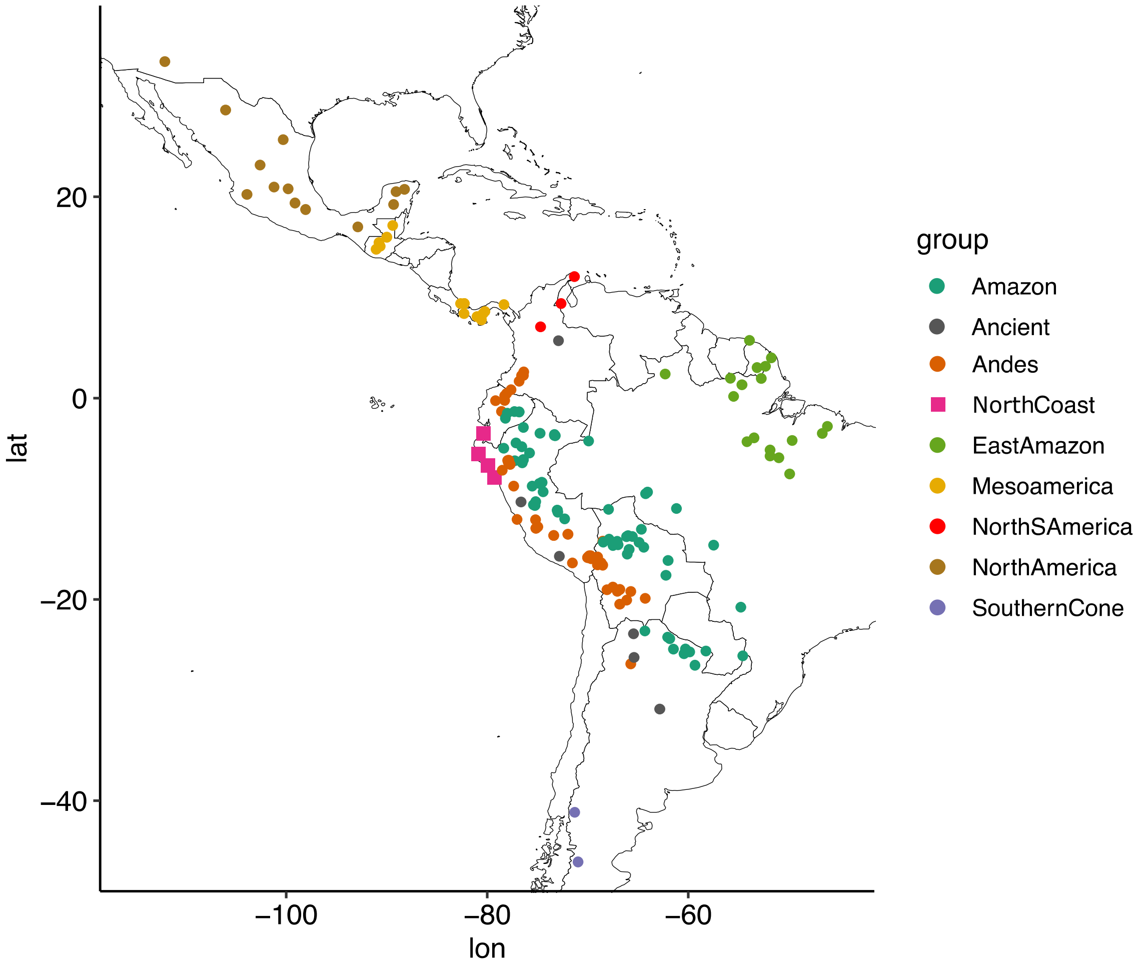

Figure S1: Locations of population samples from the whole Y chromosome continental dataset, consisting of 17 STR loci. The four North Coast populations from the present study are marked with squares.

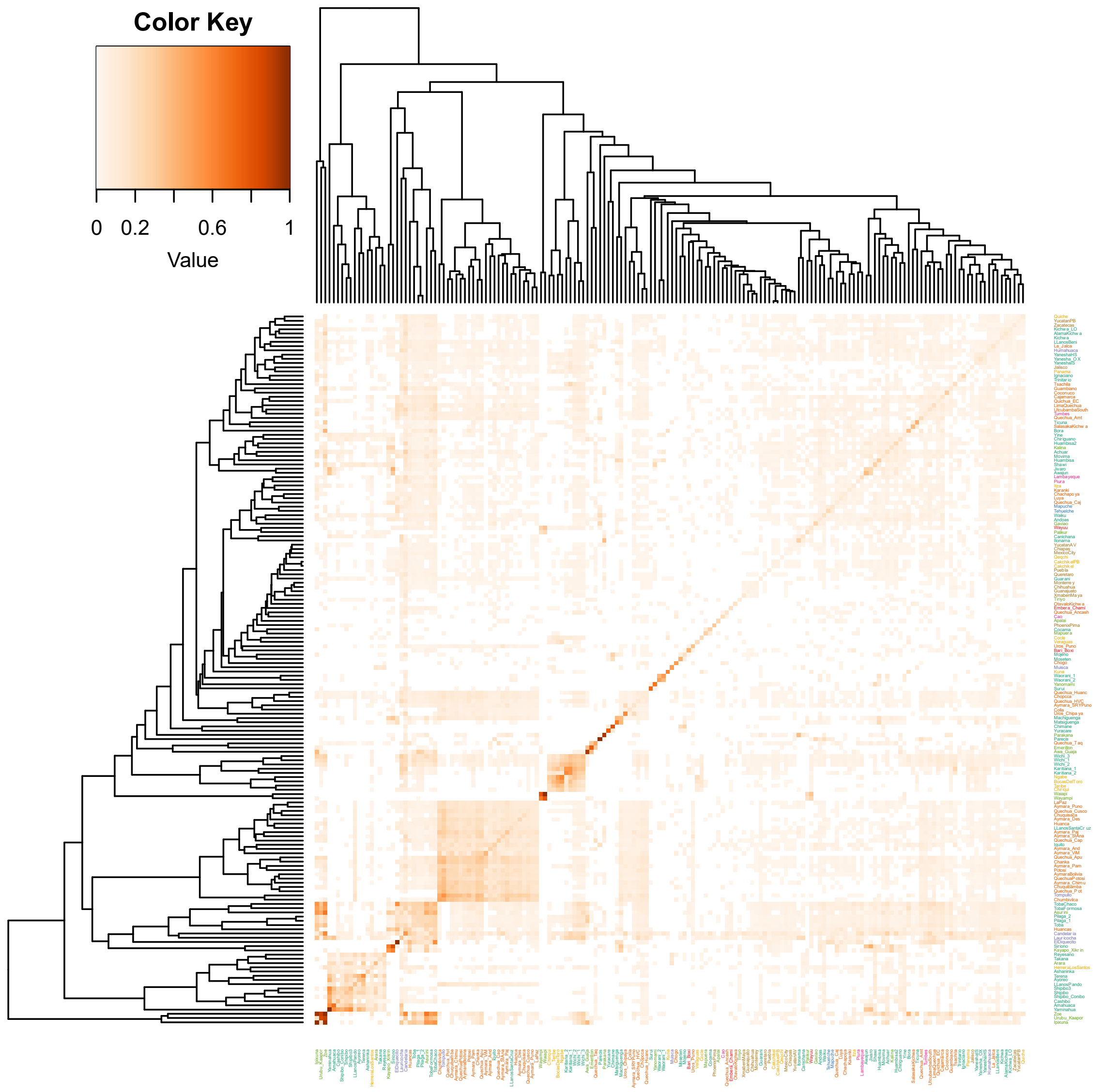

Figure S2: Heatmap showing the frequency of sharing of Y chromosome tMRCA below 2000 y between all pairs of populations in the continental dataset, adjusted for population size. The dendrogram was generated using a hierarchical clustering algorithm. The population names are colored according to their region membership as defined in Figure S1.

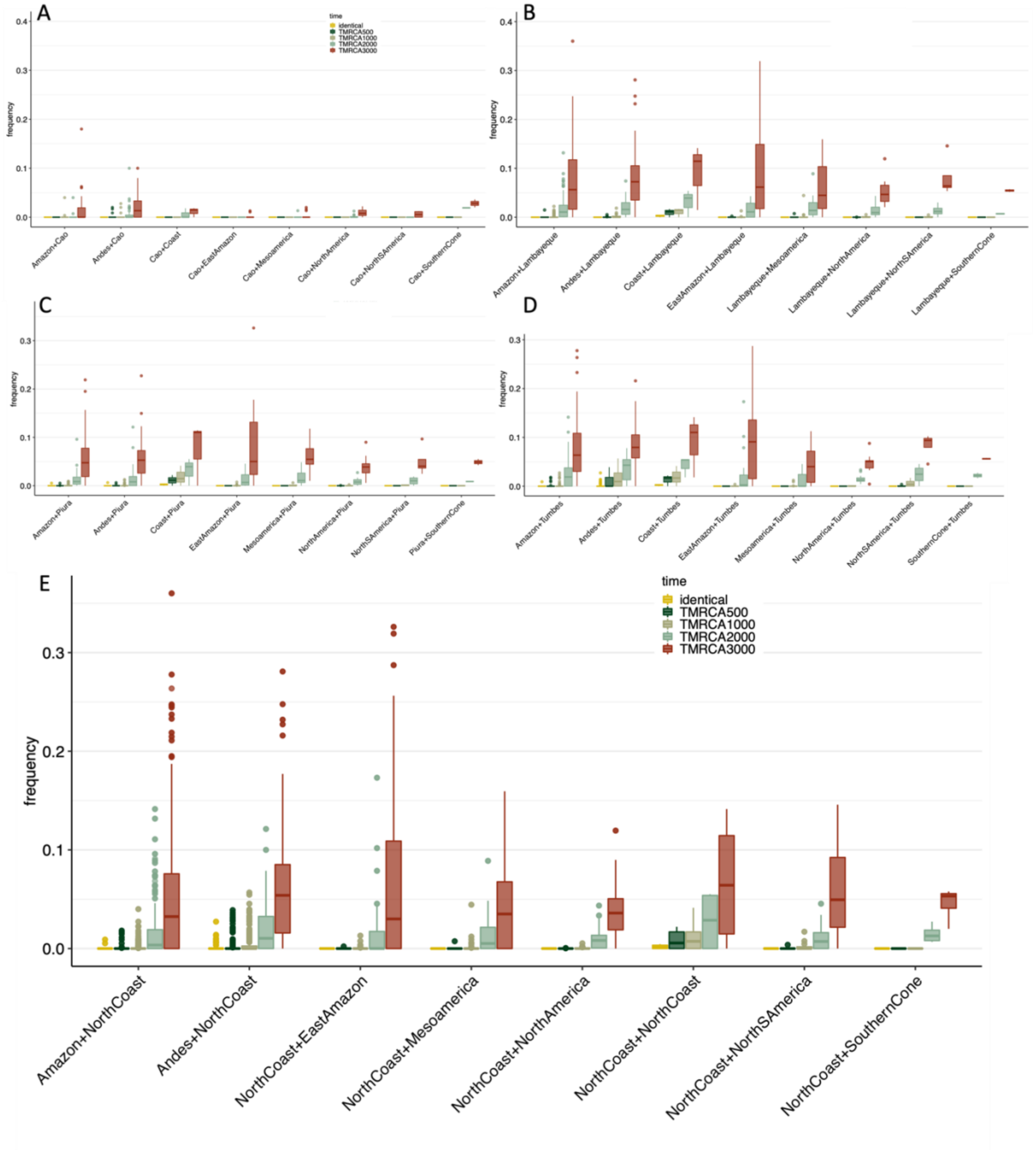

Figure S3: Boxplots of the frequency of pairs of populations with identical Y chromosome haplotypes and with an estimated common ancestor within different temporal thresholds: 500, 1000, 2000 and 3000 years ago. Panels A through D show the frequencies between one Coast population and remaining individuals from North Coast and other regions: A: Cao, B: Lambayeque, C: Piura and D: Tumbes. Panel E shows the frequencies between the whole North Coast region and other regions.

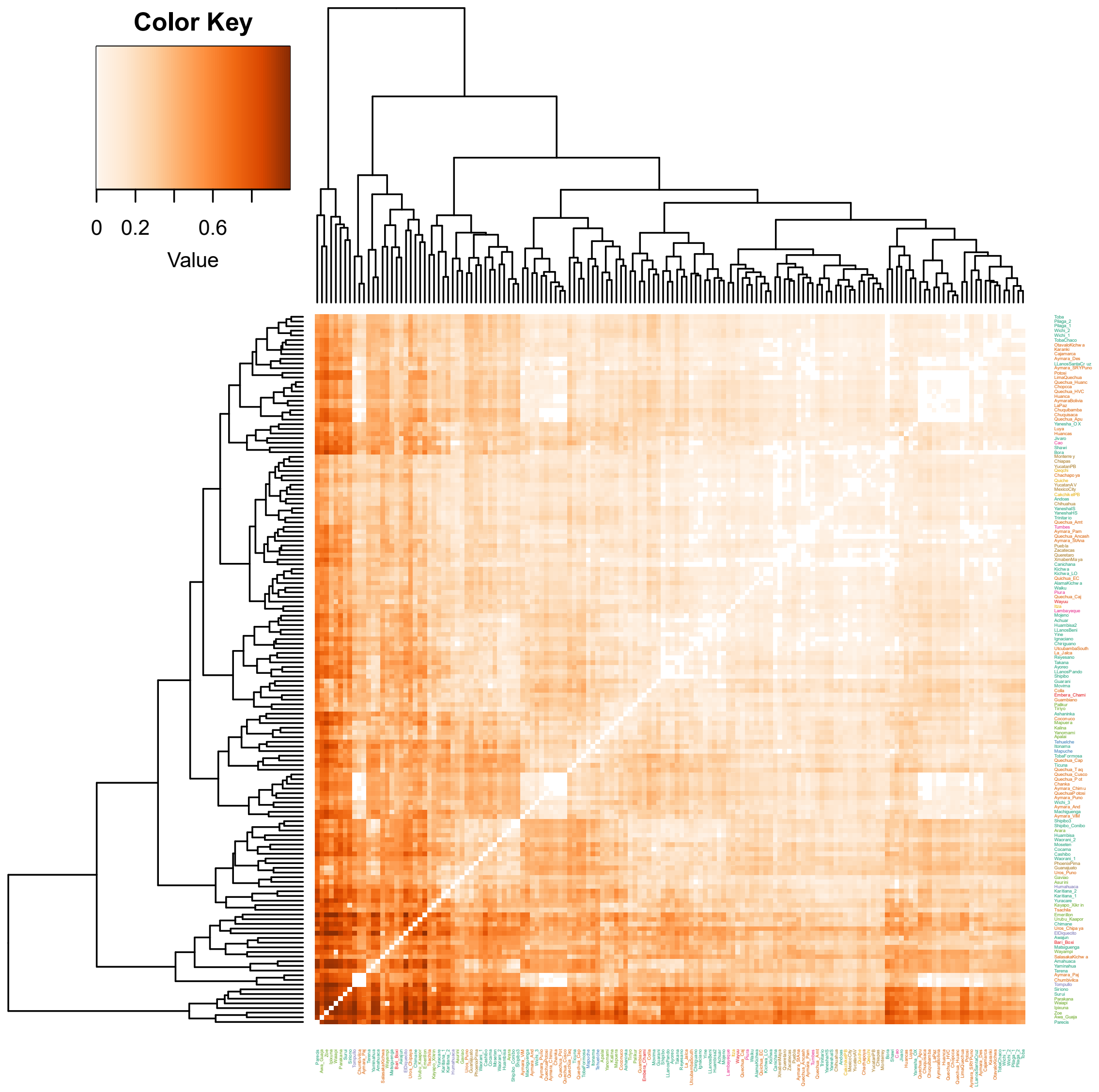

Figure S4: Heatmap showing the Y chromosome R_{ST} distances between all pairs of populations in the dataset that have at least 4 complete 17 STR individual haplotypes, and a dendrogram generated using a hierarchical clustering algorithm. The population names are colored according to their region membership as defined in Figure S1.

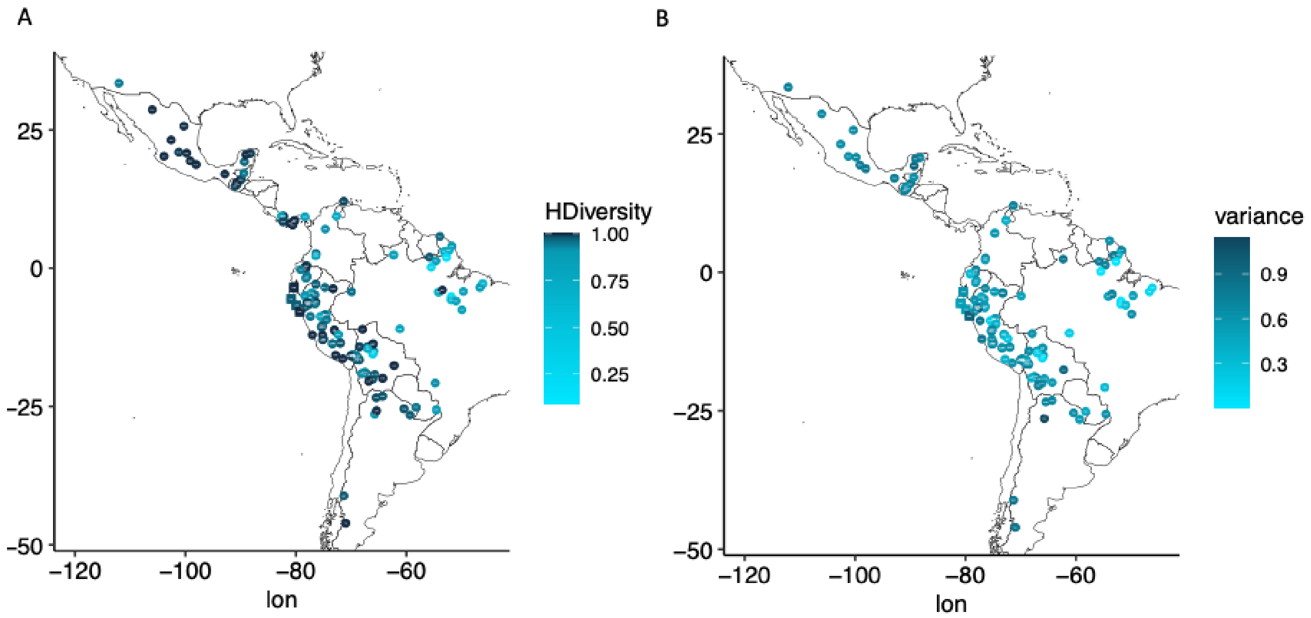

Figure S5: Population diversity for the Y-STR dataset. A. haplotype diversity. B. haplotype variance. The four North Coast populations from the present study are marked with squares.

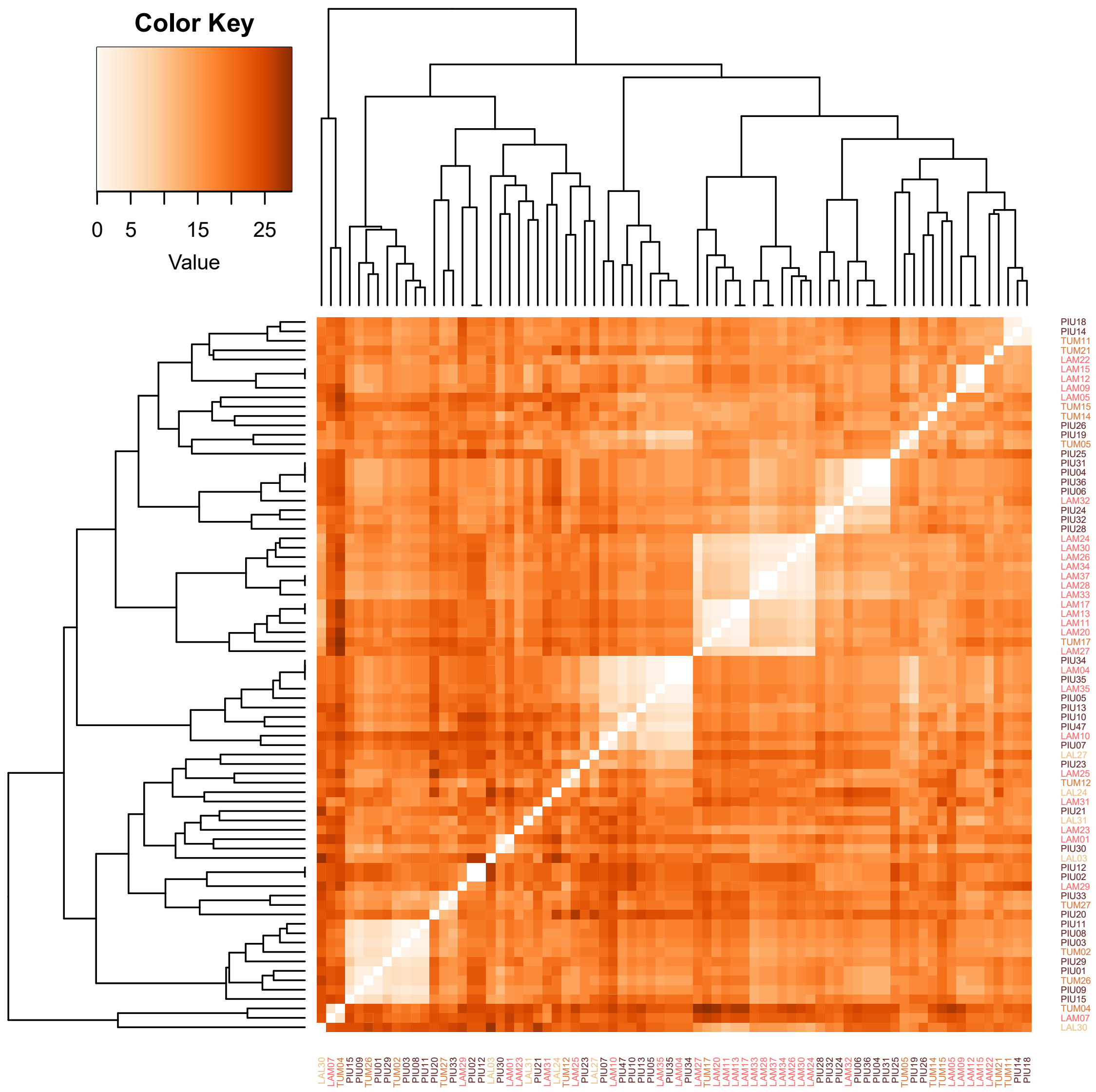

Figure S6: Heatmap based on the full 23 loci profiles of the Y chromosome, showing the distance between North Coast individuals in number of mutation steps. The individuals are colored by population (PIU: Piura, LAM: Lambayeque, TUM: Tumbes, LAL = Cao, province of La Libertad).

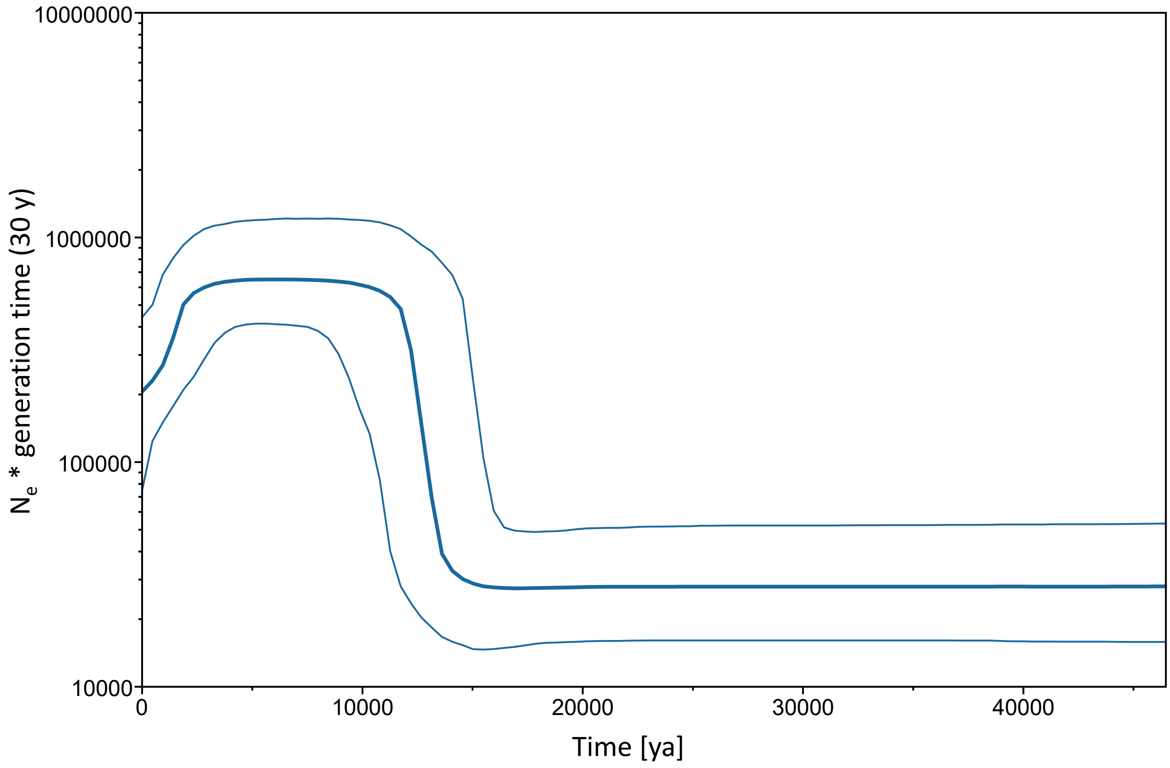

Figure S7: Bayesian Skyline Plot (BSP) generated with all the mtDNA genomes from the North Coast samples. The y axis is on a log scale and shows N_e * 30 years generation time.

Figure S8 (figure on next page): NJ trees of all mtDNA samples in the dataset. Panels A-D show haplogroups A-D, respectively. Each sample is colored by its region of origin according to the legend above. The trees are rooted with the RSRS haplotype as outgroup.

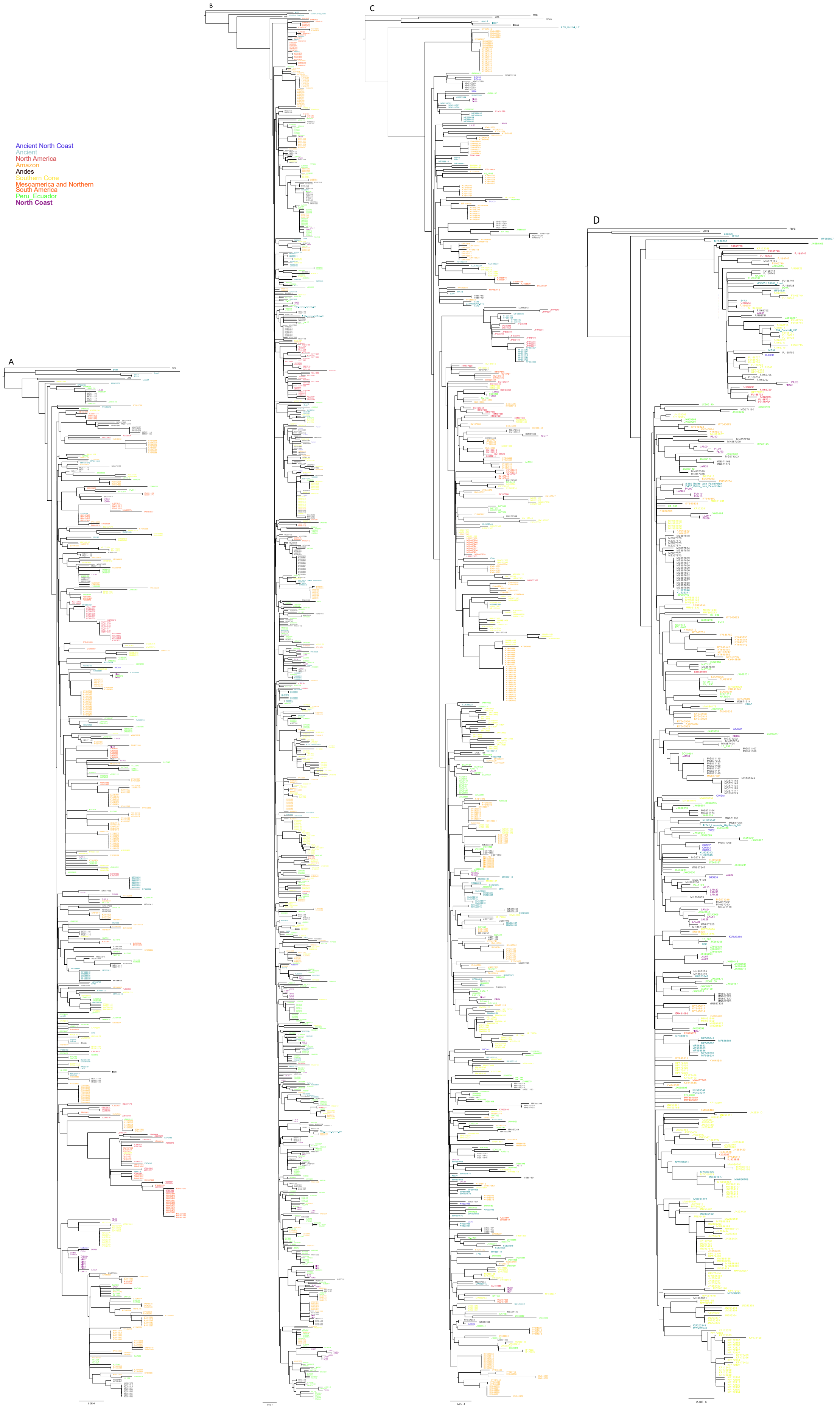
A
B
C
D
Ancient North Coast
Ancient
North America
Amazon
Andes
Southern Cone
Mesoamerica and Northern South America
Peru_Ecuador
North Coast
